# Comment on: Incidence of blindness in a population of rheumatic patients treated with hydroxychloroquine

**DOI:** 10.1093/rap/rkz041

**Published:** 2019-10-18

**Authors:** Anil Pareek, Ravi Tejraj Mehta, Shruti Dharmadhikari, Kumar Naidu

**Affiliations:** Medical Affairs and Clinical Research, Ipca Laboratories Limited, Mumbai, India

Sir, We have read the article [1] with interest and appreciate the authors’ efforts in examining the incidence of ocular toxicity in rheumatic patients treated with HCQ. HCQ continues to be an important drug in rheumatic conditions, and its use is emerging in conditions such as metabolic disorders and oncology [2]. Hence, proper understanding of ocular toxicity with HCQ is essential. Diabetes [3], hypertension [4] and chronic kidney disease [5] are common co-morbidities with rheumatic diseases. To our knowledge, this is the first study to assess the effect of such co-morbidities affecting the retina and vision, and we compliment the authors for enhancing this perspective.

With this background, it is important to note that recent recommendations on screening for chloroquine and HCQ retinopathy by the American Academy of Ophthalmology [6], the clinical guidelines by the Royal College of Ophthalmologists [7] and a study by Melles and Marmor [8] did not consider the role of co-morbidities while assessing the ocular toxicity of HCQ. This might be the reason for the higher incidence of HCQ retinopathy in the above study [8], which formed the predominant basis of the screening recommendations.

In this retrospective longitudinal study [1], the authors identified 2867 rheumatic patients from 1999 to August 2017 who had a prescription written for HCQ. Of the total 31 patients with a diagnosis of blindness or toxic maculopathy, the majority of cases were associated with co-morbidities, and only three cases had HCQ-related retinal toxicity (person-time incidence rate of 0.18 cases per 1000 person-years), each without blindness or functional vision loss. In all three patients, HCQ had been used for >18 years. It is important to note that in two patients, the daily dose exceeded the maximal recommended dose (7.2–8.1 and 7.3–8.2 mg/kg/day). The cumulative dose in these three patients was 2880, 2592 and 3600 g.

The findings of this retrospective study [1] provide reassurance regarding the safety of HCQ in routine clinical use, because the incidence of HCQ-induced retinal toxicity is rare when the maximal recommended daily dose is not exceeded. Moreover, with the availability of improved modalities, such as Spectral Domain Optical Coherence Tomography, retinal toxicity can be picked up early, ensuring safe use of HCQ.

The authors have reported that of the 31 patients, 6 patients had either type or type 2 diabetes mellitus. We request the authors to share the following additional data: how many patients out of the total population of 2867 had type 2 diabetes mellitus at baseline; the mean duration of type 2 diabetes; and the incidence of non-proliferative diabetic retinopathy in them, if any. We would also like to know the HCQ exposure (duration of use, cumulative dose and dose at initiation by actual body weight) in these type 2 diabetes patients and those who had non-proliferative diabetic retinopathy. This information would help in understanding the interplay between co-morbid type 2 diabetes in rheumatic patients and HCQ retinopathy.

*Funding*: The authors did not receive any funding for this work.

*Disclosure statement*: A.P., R.T.M., S.D. and K.N. are employees of Ipca Laboratories Limited, Mumbai, India, which manufactures and markets HCQ.
